# Post-mortem feasibility of dual-energy computed tomography in the detection of bone edema-like lesions in the equine foot: a proof of concept

**DOI:** 10.3389/fvets.2023.1201017

**Published:** 2024-01-04

**Authors:** Jolien Germonpré, Louis M. J. Vandekerckhove, Els Raes, Koen Chiers, Lennart Jans, Katrien Vanderperren

**Affiliations:** ^1^Department of Morphology, Imaging, Orthopedics, Rehabilitation, and Nutrition, Faculty of Veterinary Medicine, Ghent University, Merelbeke, Belgium; ^2^Department of Pathobiology, Pharmacology, and Zoological Medicine, Faculty of Veterinary Medicine, Ghent University, Merelbeke, Belgium; ^3^Department of Diagnostic Sciences, Faculty of Medicine and Health Sciences, Ghent University Hospital, Ghent, Belgium

**Keywords:** DECT, bone marrow edema, bone bruise, bone contusion, horse, virtual non-calcium (VNCa), veterinary medicine

## Abstract

**Introduction:**

In this proof-of-concept study, the post-mortem feasibility of dual-energy computed tomography (DECT) in the detection of bone edema-like lesions in the equine foot is described in agreement with the gold standard imaging technique, which is magnetic resonance imaging (MRI).

**Methods:**

A total of five equine cadaver feet were studied, of which two were pathological and three were within normal limits and served as references. A low-field MRI of each foot was performed, followed by a DECT acquisition. Multiplanar reformations of DECT virtual non-calcium images were compared with MRI for the detection of bone edema-like lesions. A gross post-mortem was performed, and histopathologic samples were obtained of the navicular and/or distal phalanx of the two feet selected based on pathology and one reference foot.

**Results:**

On DECT virtual non-calcium imaging, the two pathological feet showed diffuse increased attenuation corresponding with bone edema-like lesions, whereas the three reference feet were considered normal. These findings were in agreement with the findings on the MRI. Histopathology of the two pathologic feet showed abnormalities in line with bone edema-like lesions. Histopathology of the reference foot was normal.

**Conclusion:**

DECT virtual non-calcium imaging can be a valuable diagnostic tool in the diagnosis of bone edema-like lesions in the equine foot. Further examination of DECT in equine diagnostic imaging is warranted in a larger cohort, different locations, and alive animals.

## 1 Introduction

Lameness is common in horses and is mostly diagnosed in the distal forelimbs (1). When the diagnosis remains inconclusive based on radiography and ultrasonography, cross-sectional imaging methods can be performed, such as computed tomography (CT) and magnetic resonance imaging (MRI) (2–8).

MRI is a validated technique and the most commonly used cross-sectional imaging modality for a wide range of pathologies in the equine foot (7, 9, 10). Hence, MRI provides a detailed image of both the osseous and soft tissue structures of the equine lower limb (9, 11–13). Moreover, MRI can be performed while both standing (low-field magnet) and under general anesthesia (both low- and high-field magnets) (14).

The use of computed tomography (CT) imaging is emerging in equine diagnostic imaging, especially since the upcoming “standing CT” technique can be performed under sedation and with a short acquisition time of only several seconds (14–18). Conventional CT imaging generates grayscale images that are a result of the differing attenuation values within the scanned tissue. However, attenuation coefficients are not unique for any given material; it is a function of the material composition, the incoming photon energy, and the mass density of the material (19, 20). This limitation can make tissue and lesion characterization challenging, particularly in the evaluation of soft tissue.

The upcoming dual-energy CT (DECT) imaging technique offers improved tissue characterization by obtaining a second attenuation measurement at a different photon energy. As a result, two attenuation coefficients for each voxel are obtained at two photon energy measurements. The ratio of these two attenuation coefficients allows the unique differentiation of tissues with differing atomic numbers, based on the energy- and element-dependent nature of X-ray attenuation (19, 21). Hence, DECT allows for the decomposition of tissues into their constituent elements, including soft tissues (22, 23). Soft tissue characterization via DECT imaging is innovative since the evaluation of soft tissue usually requires an MRI. Therefore, DECT has allowed a new approach in human diagnostic imaging, including the detection of bone marrow edema (24–28).

Bone marrow edema was first described by Wilson et al. (29) to define the pathological appearance of bone marrow on MRI in painful human joints for which no specific radiographic abnormalities were detected (29). Bone marrow edema is an umbrella term for various histopathologic findings that cause the accumulation of fluid within the bone marrow, including hemorrhage, necrosis, fibrosis, and infrequently “true” edema (30–32). Therefore, the term “bone marrow edema-like lesion” is often preferred in human diagnostic imaging (31, 33). In the horse, the navicular bone consists of compacta and spongiosa without a medulla; therefore, the term “bone edema-like lesion” was chosen in this study. In equine diagnostic imaging, the presence of bone edema-like lesions on MRI was observed in cases where osseous injury of the distal limb was reported while the radiographs were unremarkable (34, 35). Moreover, Mizobe et al. (34) found that the presence of bone edema-like lesions on MRI has been clinically significant since the application of appropriate care based on their presence would contribute to the prevention of further injury.

The mechanism behind the detection of bone edema-like lesions differs between MRI and DECT. On MRI, bone edema-like lesions demonstrate a typically altered signal as a result of the increased water content, characteristically generating a low and high signal intensity on respectively the T1- and T2-weighted sequences, with a hyperintense signal in fat-suppressed sequences, such as the short-tau inversion recovery (STIR) sequence (28, 36–39). The STIR sequence provides homogenous fat suppression, which improves the contrast ratio between high-fluid bone edema-like lesions and the physiological, fat-rich yellow bone marrow. Additionally, water/fat separation can be achieved by the XBONE sequence; this is a gradient echo-type sequence that generates two sets of images, with one containing only the fat signal and the other containing only the water signal. On the contrary, DECT allows the detection of bone edema-like lesions via a three-material decomposition algorithm (22, 40). This algorithm subtracts the calcium from the cancellous bone, generating virtual non-calcium (VNCa) images to evaluate the fat and water components within the bone marrow.

The added value of DECT in the detection of bone edema-like lesions has already been thoroughly evaluated at different human body sites, including knee (40, 41), wrist and hand (26), ankle (42), hip (43, 44), and spine (45–47). DECT has been found to be an accurate technique in humans for the detection of bone edema-like lesions, with sensitivity, specificity, and accuracy values of, respectively, 81–94, 91–98, and 90–91% in comparison with the gold standard MRI (25, 28, 46–49). However, this diagnostic accuracy is considered to be reduced by the presence of bone sclerosis since it has been reported to be indistinguishable from bone edema-like lesions in some cases (50).

Despite the great benefits of DECT imaging in human medicine and the increasing availability of compatible refurbished CT scanners in veterinary medicine, the implementation of DECT in equine diagnostic medicine in the detection of bone edema-like lesions and other soft tissue lesions has not been investigated. The objective of this study is twofold: first, to test the hypothesis that DECT VNCa imaging is a feasible diagnostic technique to detect bone edema-like lesions in the equine foot in agreement with MRI, and second, to contribute to the further optimization of the DECT protocol for the equine distal limb.

## 2 Materials and methods

### 2.1 Material collection

In total, five unfrozen feet were collected from five different horse cadavers that were euthanized for reasons unrelated to this study. Two feet were selected based on the pathology of the foot; these feet were collected from horses that were euthanized due to an injury to the equine foot with a poor prognosis. Three other feet were collected randomly as reference feet. Each foot was removed from the fetlock joint. The post-mortem interval between euthanasia and diagnostic imaging was noted for each foot. During this interval, the material was kept refrigerated for up to a maximum of 6 h prior to diagnostic imaging. For each foot, the description of the cadaver (age, sex, and cause of death/euthanasia) was noted.

### 2.2 Diagnostic imaging

#### 2.2.1 Computed tomography

The DECT acquisition was performed using a 320-slice single-source CT scanner (Canon Aquilion ONE Vision Edition, Canon Medical Systems, Tochigi, Japan). Each foot was placed parallel to the z-axis in the isocenter of the gantry in lateral recumbency. First, the tube current for the DECT acquisition was determined via automated tube current modulation through the acquisition of an initial helical conventional CT scanogram. The DECT protocol was performed by acquiring two sequential volume scans: a low (80 kV) dataset and a high (135 kV) dataset at a rotation time of 1.5 s for both. The tube current-rotation time product was noted for each foot. The volume DECT scan acquisition time was a standard set time of 3.6 s with a scan length of 16 cm for all feet. In between the datasets, the positioning of the limbs remained unchanged, and volume scans were centered on the navicular bone. The CT dose index and dose-length product were noted for each foot.

##### 2.2.1.1 Image reconstruction

DECT images were created on the workstation via post-processing software that was made available by the vendor. VNCa images (multiplanar reformations, slice thickness of 0.5 mm) were obtained using three-material decomposition software to differentiate calcium, fat, and water. The dual-energy gradient for calcium was set at 0.70, and material formulas for fat and water were, respectively, −136/−106 and 0/0 (80 kV/135 kV). The low and high kilovoltage datasets were automatically reconstructed into conventional CT images (bone and soft tissue kernel), with a slice thickness of 0.5 mm and sent to PACS.

#### 2.2.2 Magnetic resonance imaging

The MR acquisition was performed using a low-field MRI (Vet-MR Grande, 0.25-T, Esaote, Italy). Each foot was individually covered with plastic coating and placed in lateral recumbency on the table, in a (human) knee coil centered on the navicular bone. Five sequences were acquired (Table 1): a 3D SST1-weighted sequence (slice thickness: 0.35 mm; TE: 9 ms; TR: 22 ms; flip angle: 30°), a 3D SST2-weighted sequence (slice thickness: 0.43 mm; TE: 10 ms; TR: 20 ms; flip angle: 50°), a transverse fast proton density-T2 sequence (slice thickness: 4 mm; TE: 25 ms; echo train length: 8; TR: 4,260 ms; flip angle: 90°; slice thickness: 4 mm), a sagittal STIR sequence (slice thickness: 4 mm; TE: 30 ms; TR: 4,340 ms; IT: 70 s; flip angle: 90°; slice thickness: 3.5 mm), and a transverse XBONE sequence (TE: 21.2 ms; TR: 1,440 ms; flip angle: 60°; slice thickness: 4 mm). For feet III and V, the 3D SST2-sequence was not included in the protocol.

**Table 1:** Overview of the findings of all feet (I–V) on DECT VNCa, MRI, CT, and histopathology.

**Foot**	**DECT**	**MRI and CT**	**Histopathology**
**I–III:** reference	Areas of high attenuation: • Directly adjacent to the cortical edge; • Linearly obliquely oriented within the proximal part of P3; • Adjacent to the proximal articular surfaces.	Areas of high attenuation on DECT VNCa corresponded to zones of high bone density on both the MR-T1 sequence and CT.	Foot II NB and P3: within normal limits.
**IV:** Chronic penetrating nail injury	**As listed for feet I-III including:** • NB: diffuse increased attenuation. • P3: mottled increased attenuation in the proximal half, decreasing gradually toward the distal tip.	**As listed for feet I-III including:** • NB: diffuse, hypointense T1/hyperintense STIR and XBONE signals. Diffuse disruption to erosion of the palmar compacta of the NB. • P3: marked hypointense T1/hyperintense STIR and XBONE signals involving the palmar surface and palmar processes.	•NB: extravasated erythrocytes, fibroblasts, and fibrous tissue in the spongiosa. Osteoclastic resorption of trabecular bone. • P3: eosinophilic material (protein-rich fluid) in dilated intra-medullary capillaries and in the interstitium of the adipose tissue.
**V:** bacterial infection of the distal inter-phalangeal joint	**As listed for feet I-III, including:** • NB: spherically shaped, decreased attenuation in the mid-plantar part of the compacta, surrounded by diffusely increased attenuation in the spongiosa.	**As listed for feet I-III, including**: • NB: cyst-like lesion in the mid-plantar compacta with sclerotic rim, surrounded by a hypointense T1 and hyperintense STIR signal. Content: mixed hypointense and isointense T1 signal, hyperintense STIR and XBONE signal, and diffuse hypodense on CT.	•NB: cyst-like lesions lined by sclerotic trabeculae adjacent to the macroscopic indentation of the plantar compacta. Content: dense, uniform fibrous tissue. Spongiosa surrounding the lesion: Extravasated erythrocytes with the proliferation of fibrous tissue and trabecular osteolysis.

NB, navicular bone; P3, distal phalanx.

#### 2.2.3 Image reading

The MRI, conventional CT (bone and soft tissue kernels), and DECT VNCa datasets were transferred to PACS and retrieved in OsiriX (v. 12.5.2, Geneva, Switzerland) for analysis. All datasets were randomized by a doctoral candidate and descriptively analyzed in agreement with two readers (European diplomats in veterinary diagnostic imaging) who were not present during material collection and data acquisition. Hence, the readers were unaware of which feet were considered references or pathologic, and were blinded to patient description, gross pathology, and histology findings. First, the DECT VNCa images for each foot were evaluated for the presence of bone edema-like lesions. Next, the MR images for the same foot were evaluated. The presence of bone edema-like lesions on DECT VNCa was based on an increased attenuation; for MRI, its presence was based on a hyperintense signal on the STIR sequence with a concomitant hypointense signal on the T1-weighted sequence (28, 36, 39). MRI was used as the gold standard for the detection of bone edema-like lesions. The bone and soft tissue kernel CT images were evaluated in addition to MRI to confirm the presence of pathology and sclerosis that might imitate bone edema-like lesions on DECT VNCa imaging.

### 2.3 Gross post-mortem dissection and histopathology

After image acquisition, the material was kept refrigerated at 2–5°C up to 24 h prior to gross post-mortem dissection and histopathological sample collection. For the feet selected based on pathology, bone samples were collected from the area where bone edema-like lesions were observed on MRI. For the third reference foot, bone samples were collected from the distal phalanx and the navicular bone. Bone samples were obtained by slicing the foot into sagittal slices using an automatic slicer. For the distal phalanx, cuboid samples were carefully collected using a manual saw in the dorsal plane of the mid-sagittal slice. For the navicular bone, the entire mid-sagittal slice was collected as a sample. Immediately following collection, the samples were fixed in a formaldehyde solution (4%) at room temperature for 1–2 weeks. Subsequently, the samples were transferred to an acidic decalcification solution. After a waiting period of 72–96 h (depending on sample volume), the samples were sliced using tweezers and a standard scalpel into block slices for histopathology. Successively, the slices were placed into individual cassettes per sample and immersed in a buffer solution (Na_2_SO_4_ 5%) prior to microtomy. The slices were stained with a standard hematoxylin and eosin stain. The histological samples were evaluated by a European diplomate in veterinary pathology, blinded from the MRI and DECT images.

## 3 Results

### 3.1 Subject description

In total, three reference feet (feet I-III) were collected from three different cadavers, for which no orthopedic abnormalities were reported. Two other cadavers (feet IV-V) were diagnosed with lameness in the foot. For all cadavers, the left front foot was collected, except for foot V, which was collected from the right hind limb. For feet I, II, IV, and V, the average post-mortem interval to imaging was 33 h (range 2–72 h). For foot III, the day of death was unknown.

Foot I was gathered from a 17-year-old mare that was euthanized following the complications of colic, and foot II from a 3-year-old gelding that was euthanized following an inguinal hernia after recent castration. Foot III was gathered from a mare with no anamnesis or description.

**Figure 1:**
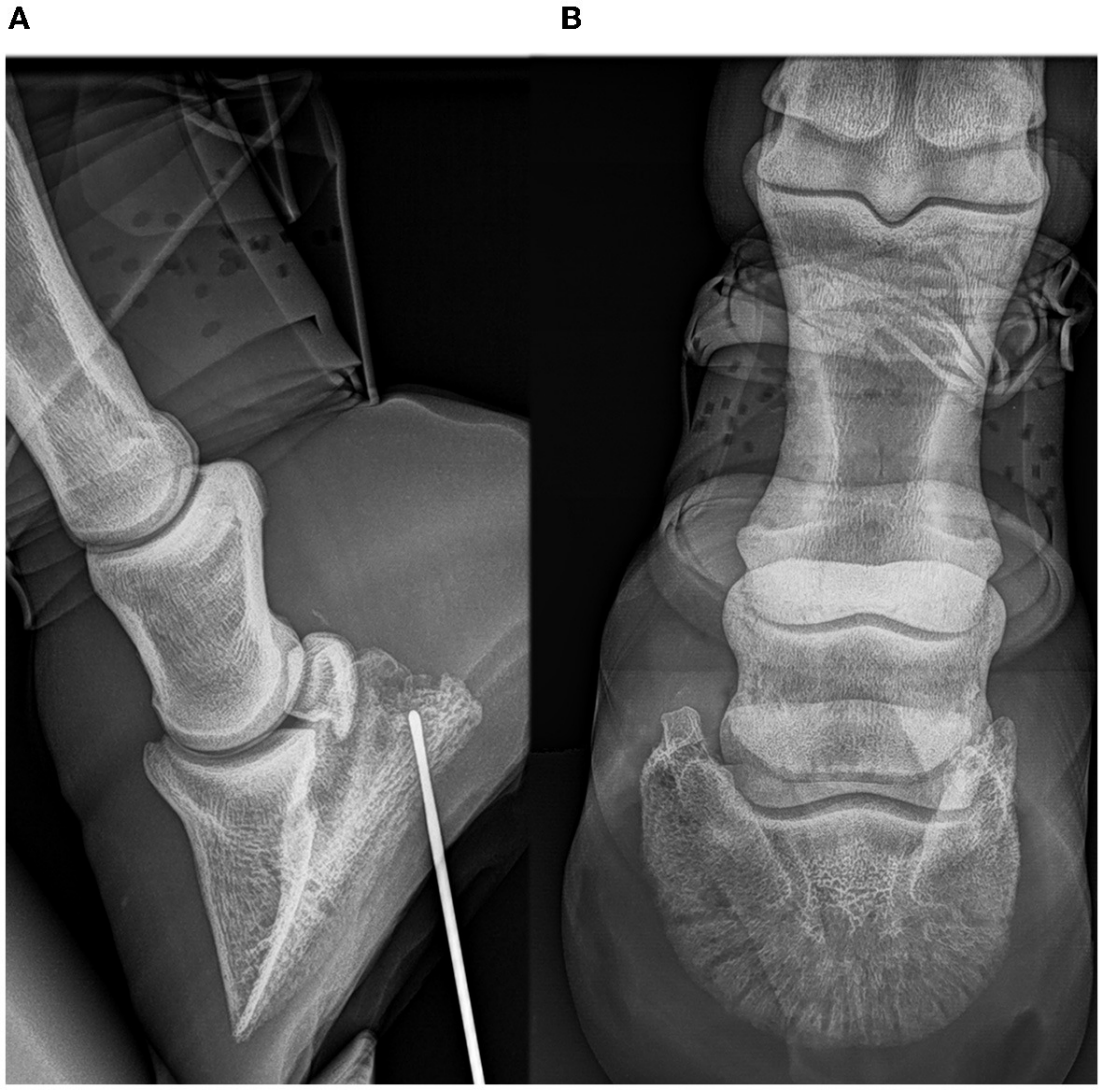
Radiographic examinations of foot IV **(A, B)**: horse with chronic penetrating nail injury of the foot sole. Lateromedial **(A)** and a dorsal 55° proximal to palmarodistal oblique projection **(B)**. A metal probe was placed in the fistula of the lateral fossa of the frog and advanced in a mildly oblique fashion in a dorsodistal to palmaroproximal direction, extending toward the navicular region.

Foot IV was gathered from a 23-year-old gelding that was euthanized because of lameness for 1 month as a result of a penetrating nail injury with infectious navicular bursitis. This was confirmed on ultrasound, bacteriology, synovial fluid of the navicular bursa, and radiographic examinations (lateromedial and a dorsal 55° proximal to palmarodistal oblique projection) (Figure 1). On the radiographs, a chronic penetrating injury of the sole was observed. A metal probe was placed in the fistula in the lateral sulcus of the frog, which advanced in a mildly oblique fashion in the dorsodistal to palmaroproximal direction, extending toward the navicular region.

Foot V was gathered from an 8-year-old Selle-Français gelding. The horse was presented with progressive, chronic lameness. On clinical examination, there was a diffuse swelling of the dorsal aspect of the coronary band of the right hind foot with a shortened stride in the walk and lameness in the straight line trot on the same foot. The lower limb flexion test of the right hind foot was strongly positive. Synovial fluid aspiration of the right hind distal interphalangeal joint revealed a small amount of amber-colored, viscous fluid. Synovial fluid analysis and bacteriology confirmed a bacterial infection. Post-diagnosis, the horse was euthanized.

### 3.2 Imaging

**Figure 2:**
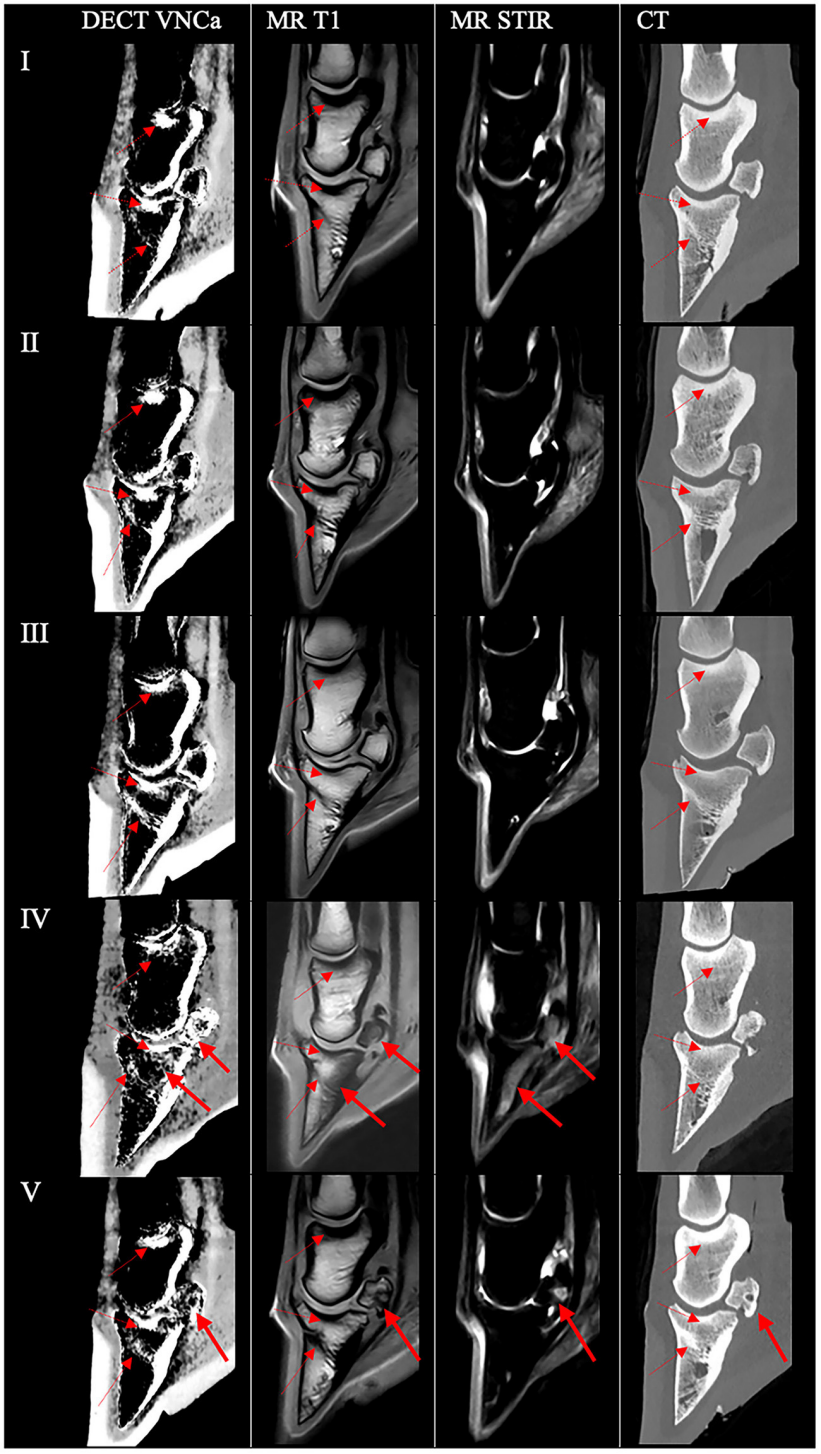
Overview of the DECT VNCa, MRI, and CT images in the mid-sagittal plane of all five feet. For each foot (left to right), the DECT VNCa image, MR T1, MR short-tau inversion recovery (STIR), and conventional bone kernel reconstructions of DECT scans are shown. In all feet, areas of high attenuation were present on the DECT VNCa image in the bone marrow/spongiosa that correspond to zones of high bone density on both the MR-T1 sequence and CT (dashed arrow), negative for bone edema-like lesions. Feet I–III were unremarkable in all modalities. (IV) In a 23-year-old horse with a chronic penetrating nail injury, DECT VNCa showed uniformly increased attenuation in the navicular spongiosa and a mottled area of increased attenuation in the proximal half of the distal phalanx, decreasing gradually toward the distal tip (full arrow), this corresponded to a hypointense T1 signal with a hyperintense STIR signal. On CT, diffuse disruption due to erosion of the palmar compacta was observed. (V) In an 8-year-old horse with chronic lameness with confirmed bacterial infection of the distal interphalangeal joint, DECT VNCa showed a spherically shaped, decreased attenuation in the mid-plantar part of the compacta, surrounded by diffusely increased attenuation in the spongiosa (bold arrow). On MRI, a cyst-like lesion in the mid-plantar compacta of the navicular bone with a sclerotic rim was observed, surrounded by a T1 hypointense and STIR hyperintense signal, indicating the presence of bone edema-like lesions. The content of the lesion generated a mixed hypointense and isointense T1 signal and a hyperintense STIR signal and was diffusely hypodense on CT (bold arrow).

A summary of imaging findings on DECT VNCa, MR, and CT and the histopathology of the three reference feet and two pathologic feet is presented in Table 1. An overview of the DECT VNCa, MR (T1 and STIR sequence), and CT images in the mid-sagittal plane of all five feet is shown in Figure 2. With a rotation time of 1.5 s, the mean tube current-rotation time product was 270 (±63) mAs (80 kV) and 111 (±23) mAs (135 kV). With a DECT scan length of 16 cm for all feet, the mean CT dose index was 24.42 (±5.21) mGy, and the mean dose-length product was 390.58 (±83.37) mGy.cm.

#### 3.2.1 Reference feet (feet I–III)

On DECT VNCa of the reference feet, the normal areas of high attenuation in the distal phalanx, navicular, and middle phalanx are presented in Table 1 and shown in Figure 2. The reference feet were unremarkable on MR and CT imaging. Areas of high attenuation on DECT VNCa imaging corresponded to zones of high bone density on both the MR-T1 sequence and CT (Figure 2).

#### 3.2.2 Foot IV

On DECT VNCa imaging of foot IV (Figure 2), besides the areas of high attenuation as in the reference feet, a uniformly increased attenuation in the spongiosa of the navicular bone and a mottled area of increased attenuation in the proximal half of the distal phalanx, which decreased gradually toward the distal tip, were present.

The MR examination showed a markedly hypointense T1 signal in the distal phalanx involving the palmar surface, extending into both lateral and medial palmar processes, with corresponding hyperintense STIR and XBONE signals. In the navicular bone, a diffuse, hypointense T1 signal with corresponding diffuse hyperintense STIR and XBONE signals was observed; this diffuse signal intensity was more pronounced slightly lateral to the mid-sagittal plane with diffuse disruption to the erosion of the palmar compacta, which was observed on both MRI and conventional CT. Focal osseous resorption of the lateral aspect of the flexor surface of the distal phalanx was present, visualized by an irregular hypointense T1 signal, hyperintense STIR and XBONE signals, and diffuse hypoattenuation with an irregular outline on CT. Moderate-to-severe distention of the navicular bursa and the distal interphalangeal joint was present.

The following soft tissue injuries and abnormalities were present: a dorsal margin lesion with focal thickening of the lateral lobe of the deep digital flexor tendon at the level of the insertion on the distal phalanx; moderate tendinopathy of the lateral lobe of the deep digital flexor tendon proximal to the suprasesamoidean region with a marked enlargement and dorsal bulging with a moderate T1 hyperintensity/hypodense lesion on CT; and thickening of the distal sesamoidean impar ligament. Additionally, a linear, small (0.3 cm in length), well-defined mineral body was present on CT that was located just proximal to the proximal ligamentous border of the navicular bone.

#### 3.2.3 Foot V

**Figure 3:**
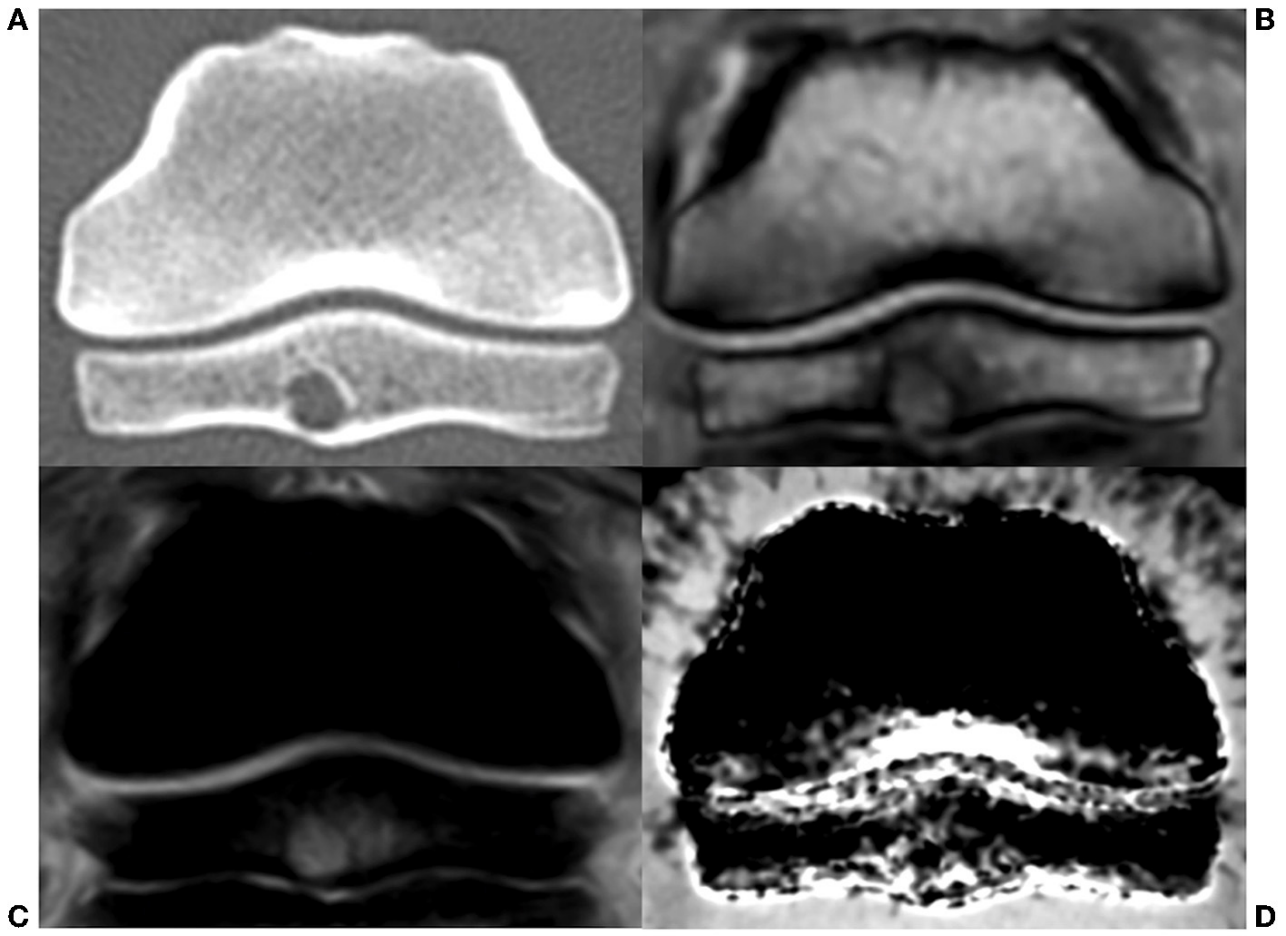
Transverse view of the navicular bone of foot V, an 8-year-old horse with chronic lameness and confirmed bacterial infection of the distal interphalangeal joint **(A–D)**. **(A)** CT: a cyst-like lesion was observed in the mid-plantar compacta with a diffuse, low-attenuating content surrounded by a smooth, sclerotic rim. **(B)** MR T1-sequence: the content of the lesion demonstrated mixed hypointense and isointense signals, surrounded by a hypointense signal. **(C)** MR short-tau inversion recovery sequence: the contents of the cyst demonstrated a mild hyperintense signal on STIR, surrounded by a hyperintense signal. **(D)** DECT VNCa: a moderate, diffuse increased attenuation was present in the spongiosa surrounding a spherically shaped decreased attenuation in the mid-plantar part.

On DECT VNCa imaging of foot V (Figures 2, 3), besides the areas of high attenuation as in the reference feet, a spherically shaped low attenuation was observed in the mid-plantar part of the spongiosa of the navicular bone, surrounded by diffusely increased attenuation in the spongiosa.

The MRI and conventional CT examinations revealed a rounded cyst-like lesion in the mid-plantar compacta of the navicular bone, extending dorsally into the navicular spongiosa and plantarly toward the navicular bursa. The content of the lesion demonstrated a mixed hypointense and isointense signal on the T1 sequence and a hyperintense signal on the STIR and XBONE sequences, surrounded by a T1 hypointense and STIR hyperintense rim (Figures 2, 3). On CT, the cyst-like lesion was 5.5 cm in diameter with a diffuse, low-attenuating content surrounded by a smooth, sclerotic rim. The flexor surface of the distal phalanx showed a diffuse, irregular outline with a demineralized aspect. Additionally, a small enthesophyte was observed at the distal margin of the navicular bone.

Evaluation of the soft tissues revealed dorsal margin irregularity of the lateral lobe of the deep digital flexor tendon, starting from just proximal to the navicular bone until the level of the proximal interphalangeal joint. The soft tissue lesions were not conclusive on soft tissue kernel CT.

### 3.3 Gross post-mortem findings and histopathology

For feet II and IV, two histological samples were taken of both the navicular bone and distal phalanx. For foot V, two samples were collected of the navicular bone. No samples were collected of feet I and III.

In foot II, no gross changes were observed. At histological examination, samples of the navicular bone were unremarkable. In the distal tip of the distal phalanx, a well-delineated, irregular focal zone of central fibrosis with a loss of adipocytes was found, rimmed by sclerotic bone trabeculae.

**Figure 4:**
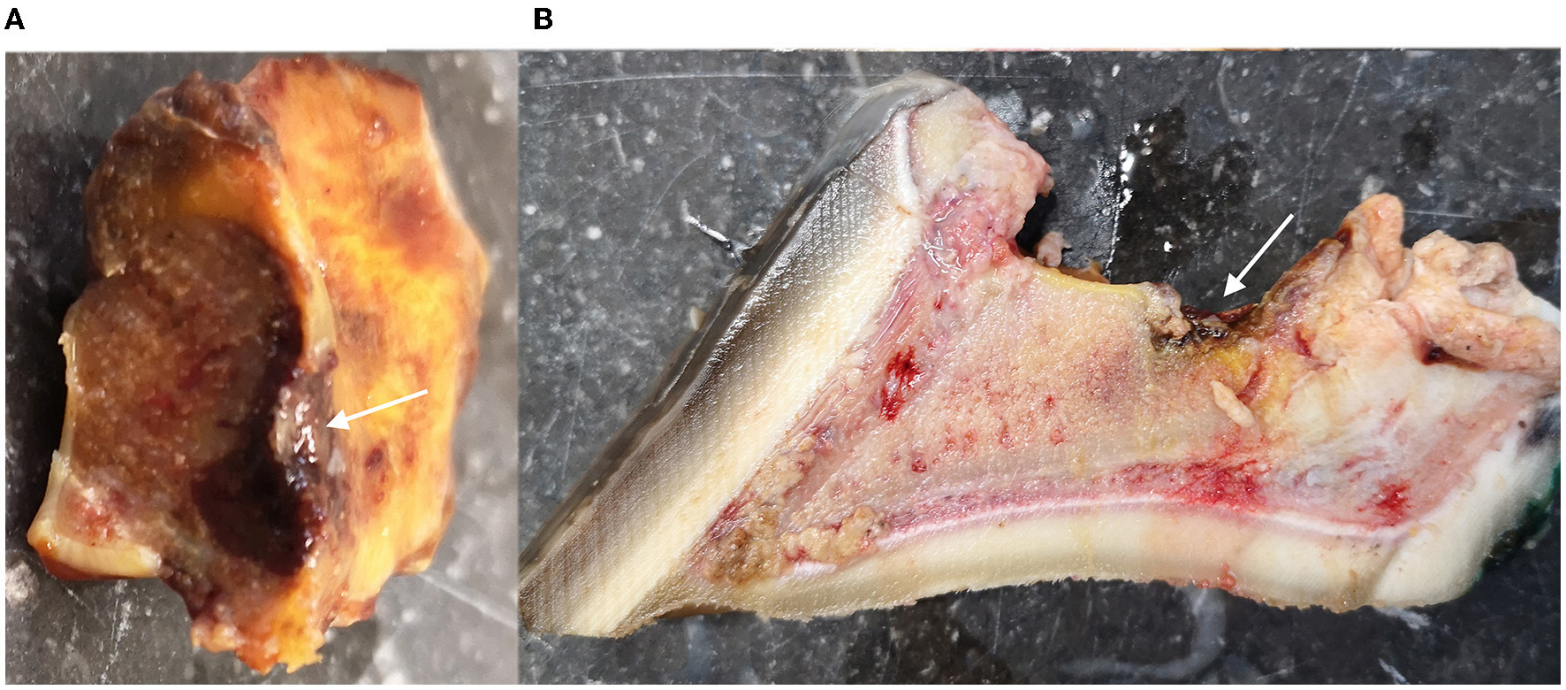
Overview of the macroscopic findings of the navicular bone and distal phalanx of foot IV, a 23-year-old horse with chronic penetrating nail injury **(A, B)**. **(A)** Mid-sagittal view of the navicular bone with diffuse indentation of the articular cartilage and the palmar compacta of the navicular bone (arrow). **(B)** Mid-sagittal view of the distal phalanx with a resorption lesion of the flexor surface (arrow).

**Figure 5:**
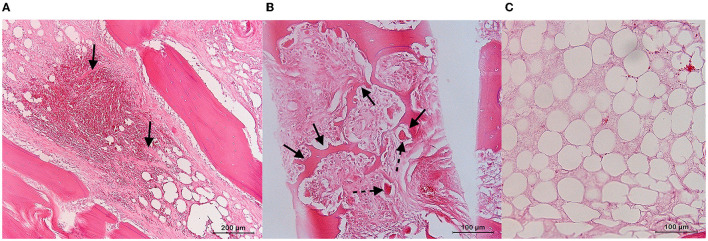
Overview of the histopathological findings of the navicular bone and distal phalanx of foot IV, a 23-year-old horse with a chronic penetrating nail injury (hematoxylin and eosin stain) **(A–C)**. **(A)** Navicular bone: intertrabecular space within a pathogenic zone showing the free red blood cells with the intervening formation of fibrous strands (arrows). **(B)** Navicular bone: multiple Howship's lacunae (bold arrow) in the bone trabeculae of the medullar space with adjacent presence of osteoclasts (dashed arrow). **(C)** Distal phalanx: increase of the interstitial space with pale eosinophilic staining (edema).

In foot IV, a diffuse indentation of the articular cartilage and palmar compacta of the navicular bone was present at gross examination, as was the hemorrhagic appearance of the underlying spongiosa (Figure 4A). For the distal phalanx, a resorption lesion of the flexor surface was observed (Figure 4B). In the histology of the navicular bone, multifocal, moderately large areas of extravasated erythrocytes were present in the spongiosa, surrounded by a mild proliferation of fibroblasts and fibrous tissue (Figure 5A). In other areas, the fibrosis was more pronounced, demonstrating densely packed fibrous tissue with osteoclastic resorption of trabecular bone as observed by the presence of multiple Howship's lacunae (Figure 5B). In the distal phalanx, infrequent lumina of intramedullary capillaries were dilated, containing lightly stained eosinophilic material (protein-rich fluid). In addition, multiple foci of expansion of the interstitium of the adipose tissue with similar material (edema) were present (Figure 5C).

**Figure 6:**
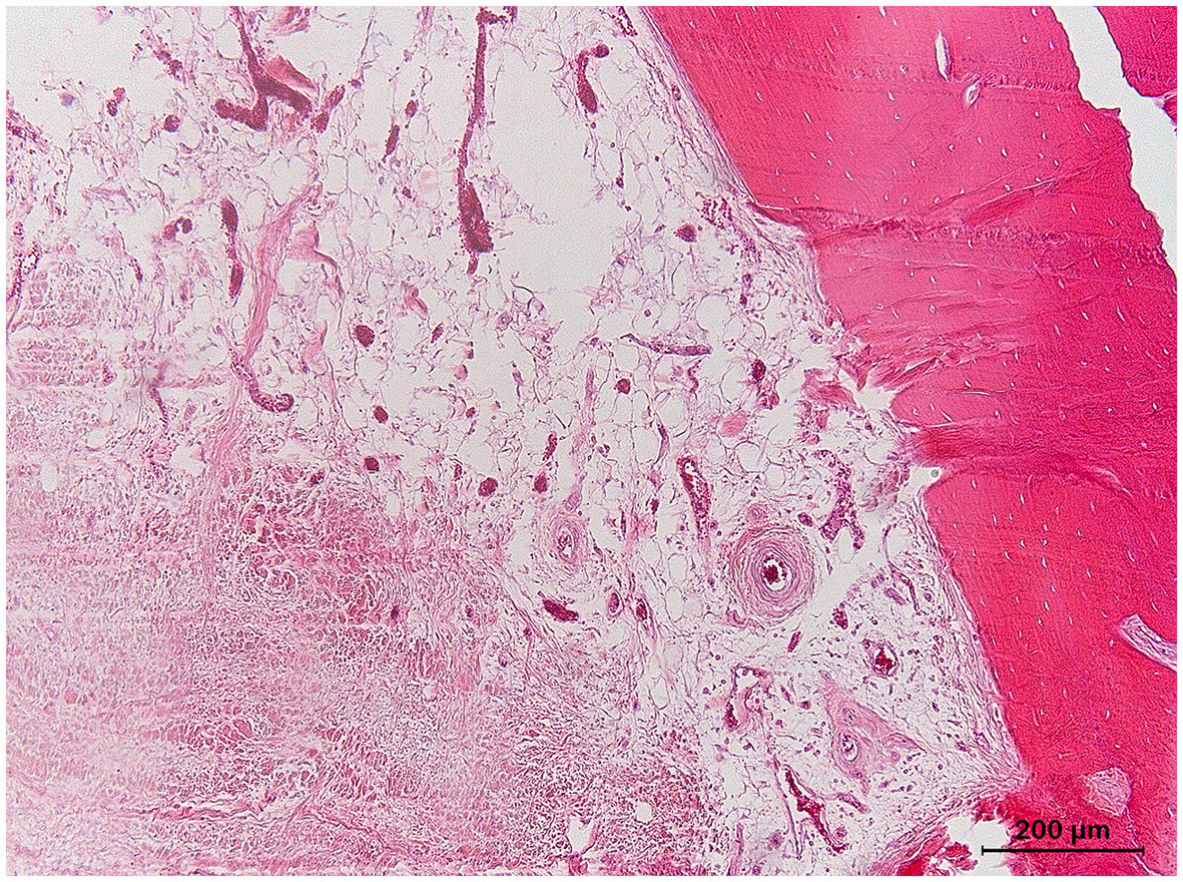
Detail of the navicular bone of foot V, an 8-year-old horse with chronic lameness and confirmed bacterial infection of the distal interphalangeal joint (hematoxylin and eosin stain). Detail of the contents of the cyst-like lesion with sclerotic rim, adipose cells in the peripheral zone, and fibrous tissue in the center.

In foot V, an indentation of the plantar compacta of the navicular bone was present at gross examination. Histologically, a cyst-like lesion lined by sclerotic trabeculae was present adjacent to this lesion. The center of the cyst consisted of dense, uniform fibrous connective tissue surrounded by adipocytes and sporadic capillaries (Figure 6). In the spongiosa surrounding the cyst-like lesion, multifocal, small areas of extravasated erythrocytes with the proliferation of fibrous tissues and trabecular osteolysis were observed.

## 4 Discussion

This initial experience with DECT imaging of the equine foot describes a feasible DECT protocol in the horse, including its appearance in both physiological and pathological cadaver feet. This study shows that DECT VNCa imaging appears to be a feasible technique for the detection of bone edema-like lesions in the equine foot. Therefore, further examination with a blinded approach in a larger cohort study is warranted to determine the accuracy of DECT VNCa in the equine foot in agreement to the gold standard, which is MRI.

In the three reference feet (feet I-III), no bone edema-like lesions were detected on MRI, which was confirmed on histology for foot II. However, on DECT VNCa imaging, high attenuation was observed in certain anatomic zones within all reference feet, for which no abnormalities were noted on MRI/CT. This observation includes the spongiosa and bone marrow directly adjacent to the cortical edge in, respectively, the navicular bone and the distal, middle, and proximal phalanx, the proximal part of the distal phalanx, and adjacent to the proximal articular surfaces in the distal phalanx and the middle phalanx (Figure 2). Similar recurring zones of high attenuation have already been described by Pache et al. (40) and Guggenberger et al. (42) in humans; an increased ratio of bone cortex to bone marrow leads to higher variance in the calcium subtraction on DECT VNCa reconstructions, caused by beam hardening and filtering effects. In this study, the high attenuation observed in the proximal part of the distal phalanx and articular surfaces is likely to be caused by physiologically high bone density, which can be observed by a hypointense signal on the MR-T1 sequence (51) and an increased attenuation on the bone kernel CT in all feet (Figure 2). These DECT VNCa artifacts appear at similar locations in the equine foot with a dual-source CT scanner (Siemens, SOMATOM Definition Flash) (unpublished data, same research team; see Supplementary Figure 1). Moreover, the role of increased cortical bone thickness was apparent in DECT VNCa images of the foot of a draft horse, in which these artifacts were more pronounced (unpublished data; Supplementary Figure 2). This breed shows a physiologically marked thickening of the cortical bone of the distal phalanx. Hence, the beam-hardening artifact was present to an increased extent in this case. Future studies need to be aware of this artifact and determine the effect of pathologically increased bone density (i.e., sclerosis) on DECT VNCa in the equine foot. The accuracy and inter-reader variability of DECT VNCa imaging should be determined for each anatomical location separately, and the diagnostic value of a DECT scan could possibly be breed-dependent. Finally, future studies cannot directly extrapolate the accuracy of DECT imaging from adult to adolescent animals since the bone marrow in these patients is still immature and undergoing red-to-yellow bone marrow conversion. Similar to MRI, the immature, red bone marrow can be wrongfully interpreted as bone edema-like lesions since both have a high water composition (52). Furthermore, horses with systemic diseases were not included in this study to avoid the presence of yellow-to-red bone marrow reconversion, which has been described in human cases as secondary to an increased physiological demand or an ongoing systemic stress reaction (53, 54).

In two feet (IV-V), bone edema-like lesions were detected on MRI, defined by a typical altered MRI signal, including a hyperintense STIR signal and a hypointense T1 signal (37). In this study, in the case of bone edema-like lesions on MRI, VNCa imaging showed a diffuse high attenuation in the affected bone (i.e., navicular bone and/or distal phalanx). The presence of bone edema-like lesions on MRI was confirmed via histopathology and corresponded to the findings described in the literature (Figures 5, 6) (30–32, 55, 56). The bone edema-like lesion area observed on diagnostic imaging consisted of normal bone marrow/spongiosa, in which there were focal zones of hemorrhage, fibrosis, foci of necrosis, osteolytic trabeculae, increased vascularization, and/or “true” edema (defined in this study as the accumulation of extracellular protein-rich fluid, as the swelling of fat cells, and by the incipient disintegration of fat cells). Of all these abnormalities that are categorized under the umbrella term “bone edema-like lesion”, there was only a selected range of the abnormalities detected in every case. Furthermore, for each case, a different abnormality dominated the histological sample: hemorrhage in foot IV, fibrosis, and “true” edema in foot V. Consequently, future studies should determine the influence of the lesion characterization on the accuracy of DECT VNCa imaging since this imaging technique solely evaluates the fat and water components within the bone marrow (22, 40). For example, in foot IV, the presence of iron in the hemorrhage zone in the navicular bone could be a determining factor for the increased attenuation of DECT (57). Histology also confirmed the presence of bone edema-like lesions surrounding the sclerotic rim of the cyst-like lesion in the navicular bone. In comparison with MRI, sclerosis and bone edema-like lesions cannot solely be differentiated on DECT VNCa imaging; therefore, the evaluation of the DECT VNCa images should be performed along with conventional CT images. Finally, the etiology of bone edema-like lesions in horses differs from that in humans. In the horse, bone edema-like lesions are often associated with osteoarthritis, soft tissue injury, acute trauma, or biomechanical stress (35), whereas in humans, they are also often seen in non-traumatic diseases, including rheumatoid arthritis and gout (26, 28, 41).

Regarding the DECT scan protocol, the tube current-rotation time product was calculated by automatic tube current modulation for each foot via an initial helical conventional CT scanogram. In this study, the mean CT dose index was 24.42 (± 5.21) mGy and the mean dose-length product was 390.58 (± 83.37) mGy.cm for a 16-cm DECT volume scan with a rotation time of 1.5 s. Although radiation dose restrictions are less strict in animals than in humans, future studies could determine the minimal requirement in rotation time per anatomical location to obtain an acceptable signal-to-noise ratio and minimize radiation dose. Currently, only limited information is available on human studies regarding radiation doses associated with multi-energy imaging compared with platforms that use dual-source technology (58). Apart from the scan parameter settings, it is crucial to position the patient or material in the isocenter of the gantry to generate the most optimal results, especially when using automated tube current modulation (59). Therefore, future studies should determine the accuracy of DECT VNCa imaging with the upcoming standing CT technique since a scanogram and automatic tube current modulation may not be feasible.

Another important consideration when evaluating DECT images is that the DECT material decomposition only works for the defined substances. Therefore, the bone tissue in the DECT image is not classified correctly. Hence, the conventional CT dataset, which is reconstructed from the DECT datasets, should still be used to evaluate the bone tissue. Additionally, future studies are advised to focus on the application of DECT on the equine lower limb (i.e., distal to the metacarpal and metatarsal region), as the accuracy of material characterization in more proximal image acquisition may be hampered by a photon starvation artifact (46). Moreover, as previously mentioned while discussing radiation dose, DECT can be performed through different techniques depending on the vendor; therefore, readers must be aware of the specific limitations of the technique they are using (60). Similar to traditional CT imaging, the scanning protocol will require small adjustments between different anatomical locations.

Certain limitations of this proof-of-concept study should be considered. Since this study was performed on post-mortem material, the appearance of bone edema-like lesions on MRI and DECT VNCa may differ in comparison with the ante-mortem equine foot. As a result of tissue autolysis, the intra-osseous STIR hyperintensity may be affected (61, 62). Therefore, the histopathology of areas where bone edema-like lesions were observed on MRI was included to confirm the presence of histopathological changes in line with bone edema-like lesions described by Zanetti et al. (31), Thiryayi et al. (32), and Plenk et al. (30). Future examinations should also include the histopathology of all reference subjects since no consensus has been reached yet on the definition of normal bone marrow in DECT (63). It has been hypothesized that differing anatomical regions or DECT technology may affect the optimal cutoff value to differentiate bone edema-like lesions from normal bone marrow on DECT VNCa; therefore, the proposed values range between −80 and 6 HU (64). Furthermore, the sample size was limited. The two feet that showed bone edema-like lesions on MRI and DECT VNCa are both of an infectious-inflammatory nature. Nevertheless, as mentioned above, bone edema-like lesions in the horse are often of a traumatic nature (35). Hence, further examinations should be made to determine whether differing etiopathogeneses affect the accuracy of DECT VNCa in the detection of bone edema-like lesions. Moreover, DECT VNCa could have been preferred compared with high-field MRI as a gold standard for the detection of bone edema-like lesions since, for low-field MRI, a lower spectral separation between fat and water has been observed, which imposes limits on the ability to perform frequency-selective fat suppression (65).

Overall, DECT VNCa imaging encompasses great advantages since it combines the advantages of high spatial resolution conventional CT with great visualization of bone structures, complemented by the ability to detect bone edema-like lesions, which was previously limited to MRI. Moreover, a range of additional images can be reconstructed via the post-processing software beyond the DECT VNCa imaging, including DECT collagen maps or virtual non-contrast images. This initial study may contribute to protocol optimization and future clinical use of DECT in veterinary diagnostic imaging.

## 5 Conclusion

In this proof-of-concept study, DECT VNCa imaging allows the evaluation of bone edema-like lesions in the equine foot. The appearance of the normal equine foot and feet with bone edema-like lesions on DECT VNCa imaging are discussed, including the most important caveats of this initial experience with DECT in the equine foot. Further examination is warranted in a larger cohort, different locations, different diseases, different gradations of lesions, and alive animals, which will simultaneously increase the experience in DECT in veterinary diagnostic imaging.

## Data availability statement

The original contributions presented in the study are included in the article/Supplementary material, further inquiries can be directed to the corresponding author.

## Ethics statement

This research was in compliance with European legislation on animal experimentation (EU directive 2010/63/EU). Formal ethical approval was waived by the ethical committee, based on Belgian and European legislation (EU directive 2010/63/EU), as all tissues were derived post-mortem.

## Author contributions

JG: wrote the article. JG, LV, LJ, and KV: design of study. JG and LV: data collection. ER, KC, and KV: analysis of data. JG, LV, and KV: interpretation of results. JG, LV, ER, KC, LJ, and KV: revised the article for intellectual content and final approval of the completed article. All authors contributed to the article and approved the submitted version.
