# Supplementary material for: Post-mortem feasibility of dual-energy computed tomography in the detection of bone edema-like lesions in the equine foot: a proof of concept
Source: Front Vet Sci. 2024 Jan 4;10:1201017. doi: 10.3389/fvets.2023.1201017 (PMC10797750; doi:10.3389/fvets.2023.1201017)
Supplement: Supplementary file 1 [file Data_Sheet_1.PDF]

Supplementary Material

Post-mortem feasibility of dual-energy CT (DECT) in the detection of bone edema-like lesions in the equine foot: a proof of concept

Jolien Germonpré[1*], Louis Vandekerckhove[1], Els Raes[1], Koen Chiers[2], Lennart Jans[3], Katrien Vanderperren[1]

[1] Department of Morphology, Imaging, Orthopedics, Rehabilitation, and Nutrition, Faculty of Veterinary Medicine, Ghent University, Merelbeke, Belgium

[2] Department of Pathobiology, Pharmacology, and Zoological Medicine, Faculty of Veterinary Medicine, Ghent University, Merelbeke, Belgium

[3] Department of Diagnostic Sciences, Faculty of Medicine and Health Sciences, Ghent University Hospital, Ghent, Belgium

*** Correspondence:**
Jolien Germonpré
Jolien.germonpre@ugent.be

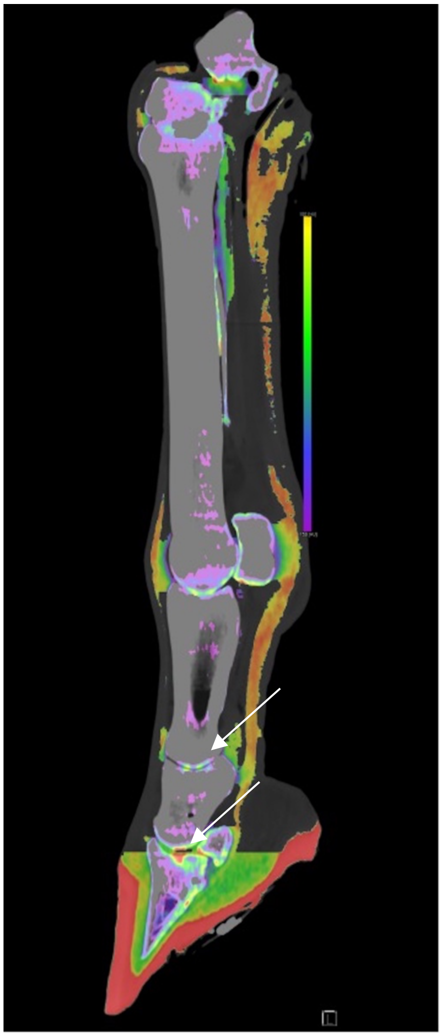

Supplementary Figure 1. Equine foot scanned with dual-source CT scanner (Siemens, SOMATOM Definition Flash). Scan parameters: 80 kV + 140 kV, Sn Filter, slice thickness 1mm, 646 mA. Zones of high attenuation as a result of increased bone density that were observed in the equine foot scanned with single-source CT scanner (Canon, Aquilon ONE Vision Edition) can also be observed in a equine foot scanned with Siemens dual-source CT scanner (arrows).

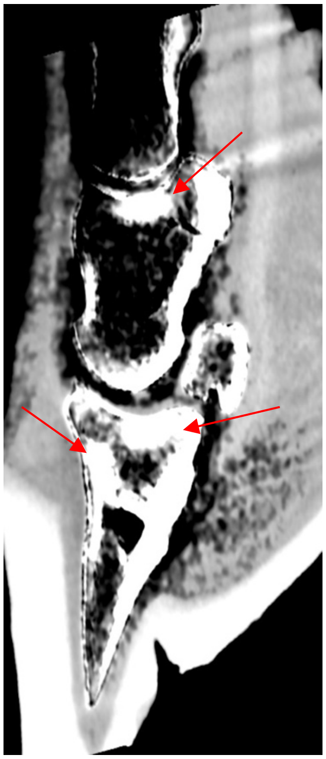

Supplementary Figure 2. Foot of a draft horse scanned with single-source CT scanner (Canon, Aquilon ONE Vision Edition). **Scan parameters:** See main article '2. Materials and methods, 2.2 Diagnostic imaging'. This equine foot was collected from a 3-year-old draft horse that acutely died due to rupture of the cecum. The role of increased cortical bone thickness was apparent in DECT VNCa images of the foot of a draft horse, in which these artefacts were more pronounced (arrows). This breed shows a physiological marked thickening of cortical bone of the distal phalanx.
